# Improved HIV case finding among key populations after differentiated data driven community testing approaches in Zambia

**DOI:** 10.1371/journal.pone.0258573

**Published:** 2021-12-02

**Authors:** Joseph Kamanga, Kayla Stankevitz, Andres Martinez, Robert Chiegil, Lameck Nyirenda, Florence Mulenga, Mario Chen, Mulamuli Mpofu, Sam Lubasi, Moses Bateganya

**Affiliations:** 1 FHI 360, Lusaka, Zambia; 2 FHI 360, Durham, North Carolina, United States of America; 3 FHI 360, Gaborone, Botswana; Johns Hopkins University, UNITED STATES

## Abstract

**Introduction:**

Open Doors, an HIV prevention project targeting key populations in Zambia, recorded low HIV positivity rates (9%) among HIV testing clients, compared to national adult prevalence (12.3%), suggesting case finding efficiency could be improved. To close this gap, they undertook a series of targeted programmatic and management interventions. We share the outcomes of these interventions, specifically changes in testing volume, HIV positivity rate, and total numbers of key populations living with HIV identified.

**Methods:**

The project implemented a range of interventions to improve HIV case finding using a Total Quality Leadership and Accountability (TQLA) approach. We analyzed program data for key populations who received HIV testing six months before the interventions (October 2017–March 2018) and 12 months after (April 2018–March 2019). Interrupted time series analysis was used to evaluate the impact on HIV positivity and total case finding and trends in positivity and case finding over time, before and after the interventions.

**Results:**

While the monthly average number of HIV tests performed increased by only 14% post-intervention, the monthly average number of HIV positive individuals identified increased by 290%. The average HIV positivity rate rose from 9.7% to 32.4%. Positivity rates and case finding remained significantly higher in all post-intervention months. Similar trends were observed among FSW and MSM.

**Conclusions:**

The Open Doors project was able to reach large numbers of previously undiagnosed key populations by implementing a targeted managerial and technical intervention, resulting in a significant increase in the HIV positivity rate sustained over 12 months. These results demonstrate that differentiated, data-driven approaches can help close the 95-95-95 gaps among key populations.

## Introduction

HIV testing services (HTS) are critical to achieve the Joint United Nations Programme on HIV/AIDS (UNAIDS) 95-95-95 targets of 95% of people living with HIV (PLHIV) knowing their status; 95% who know their status on treatment; and 95% on treatment virally suppressed [1]. While substantial progress has been made, HIV case finding among key and other priority populations remains challenging [2, 3]. New, innovative approaches are required to reach HIV-positive individuals, especially those who have challenges accessing facility-based testing or have never previously been tested.

Reaching key populations (KPs)—including men who have sex with men (MSM), transgender persons (TG), and female sex workers (FSWs)—with HIV prevention and testing services is a crucial last mile to ending the HIV epidemic [2]. In 2018, more than half of new HIV infections among adults worldwide occurred among KPs and their partners [4]. Many factors, often stemming from criminalization of certain behaviors, make it difficult for KPs to access facility-based testing services [5]. Yet, until recently, most HTS were delivered through facility-based settings.

Several approaches have been developed to improve access to HIV testing for individuals not reached through traditional facility-based services, including home-based, mobile, index (testing of sexual partners and family members), social network, and self-testing [6–8]. Uptake and positivity rates of these approaches vary among different populations [8]. A recent systematic review found that while community-based testing modalities resulted in lower HIV positivity rates in the general population, positivity rates among KPs were high, from 24% to 28% [6]. Index testing and social network strategy (SNS) have also demonstrated high positivity rates among KPs [9, 10]. However, concerns have been raised regarding index testing, especially with respect to confidentiality and client rights [11]. Thus, a mix of interventions is needed. The effectiveness of the strategies depends on the context of the local epidemic, existing gaps in testing coverage, and the needs of the target populations [12].

In Zambia, key populations have a high burden of HIV, yet there is limited data available due to the criminalization of sex work and homosexuality in the country [13]. In 2018 PEPFAR estimated that prevalence of HIV was 41.6% among FSW and 17.1% among MSM [14].

From July to September 2017, the Open Doors Project (ODP) in Zambia reported a positivity rate of 9% among KPs reached by the project, substantially lower than the estimated prevalence in these populations, as well as the Zambia national adult prevalence of 12.3% [15]. The low positivity rate was an indication that testing approaches implemented prior to September 2017 were not effective or were not deployed with fidelity. In response, the project undertook a review of strategies and technical approaches used and planned remedial actions.

We describe the design and implementation of an improved testing and linkage approach and the associated changes in number of KP individuals tested (volume tested), proportion who tested positive (positivity rate), and number who test positive (case finding rate). Results from this real-world approach to improving HIV testing efficacy will help other HIV testing providers adapt the large number of testing modalities and approaches to their own contexts.

## Methods

### Setting and population

The USAID Open Doors project is funded by the U.S. President’s Emergency Plan for AIDS Relief through the United Stated Agency for International Development (USAID). The ODP is the largest KP project in Zambia and is implemented by FHI 360 and the Zambia Health Education and Communications Trust (ZHECT), a local nongovernmental organization, in collaboration with several KP-led civil society organizations (CSOs). The project works in 8 high HIV burden districts (prevalence range 6.9%-16.1%), with major centers of economic activity and transit routes within five provinces, as shown in Table 1.

**Table 1:** Provinces and districts supported by the ODP.

Province	HIV Prevalence (2018) [14]	Districts supported by ODP
Central	12.6%	Kabwe and Kapiri Mposhi
Copperbelt	14.7%	Kitwe and Chililabombwe
Lusaka	16.6%	Lusaka Urban and Chirundu
North Western	6.3%	Livingstone
Southern	12.5%	Solwezi

The ODP has operated since May 2016. With an implementation design based on Zambia National HIV and AIDS Strategic Framework for HIV testing services (HTS) [16], the program aims to successfully identify KP, offer HTS, and link those who test negative to prevention interventions and those who test positive to ART. The project has supported testing at project facilities (e.g., wellness centers) and in the community through outreach.

### Intervention

To improve fidelity in case identification, ODP applied the Total Quality Leadership and Accountability (TQLA) intervention, an FHI 360 adaptive management approach [17]. TQLA is a strategy for improving leadership, strengthening data collection systems, and improving utilization of data to improve outcomes.

The core principles of the TQLA approach are adaptive leadership and regular review of data. Project leaders and staff convene meetings with site-level health care workers to discuss and prioritize project challenges and co-create solutions. After key interventions are agreed upon, program staff put in place a plan to meet regularly to review data, identify gaps, and continue to implement solutions. These regular data reviews, also called Situation Room Meetings (SRM), entail review of granular, site-level data on a daily or weekly basis to inform where technical assistance (TA) should be targeted. TA is provided over the phone or in person.

To address the gaps identified in case identification in the OPD project, outreach workers from the eight project districts and representatives from the KP community were engaged in March 2018. Interventions to improve case identification were adopted, as summarized in Table 2. KPs were engaged as peer promoters and were trained and directly supported implementation of SNS and index testing approaches at community level.

**Table 2:** Strategies used to improve HIV positivity rate.

Strategy	Description
Hot spot mapping and targeting	Analyzed existing “hot spots” (i.e., bars/taverns, brothels, shebeens, streets, nightclubs) and places where KPs congregate by KP type to determine HIV test positivity during the three months prior to intervention (January–March 2018) Applied the 80/20 rule to identify the top 20% of hot spots responsible for 80% of all identified HIV-positive KPs during the prior three months. These were largely hot spots with a combination of greater KP population and higher HIV positivity [18]. Revised microplans and site maps to highlight priority hot spots and characteristic features of KPs (size, type, place, and time of congregation), and determined appropriate HTS approaches and scheduling activities to reach KPs (daytime, nights, etc.) Conducted daily outreach to hot spots generating the greatest number of positives
Moonlight testing	Adopted moonlight HIV testing to reach individuals who were hard to reach during daytime testing hours
Risk assessments prior to testing	Trained and deployed peer promoters and lay counsellors to use risk assessment tools to identify and prioritize HIV testing for KP individuals likely to test positive Trained and deployed peer promoters and counsellors to classify KPs as either MSM, FSW, or transgender using the PEPFAR standard KP classification tool [11]
Enhanced peer outreach approach (EPOA) training	Peer educators trained on demand creation and reaching hard-to-reach KP members [19]
Index case testing (ICT)/partner notification services (PNS), bridge index testing	Trained and deployed peer promoters and counsellors to adopt ICT/PNS to target HTS to partners of index cases Offered HTS to sexual partners of each index client Offered HTS to male partners of index FSWs (referred to as "bridge" clients) as well other FSWs they identified through PNS
Social network strategy (SNS)	Integrated HTS into existing social network activities, where the index client introduces friends within her/his social network to access HTS. “Friends” were not necessarily sexual partners of the index client, but merely belonged to the same social network.
HIV self-test	Distributed self-test kits through index clients to their partners and within social networks to reach hidden and hard-to-reach KPs Those who tested HIV positive using self-test kits received an HIV confirmatory test prior to linkage to ART

Consequently, the ODP introduced daily (Monday-Friday) SRMs project wide, and used the framework to evaluate district performance, pivot resources and cascade accountability throughout the team. District and community teams reported data daily, on key indicators (# tested, # declined test, # known HIV-positive status, # positive, # linked to treatment) which were aggregated, visualized, and the trends analyzed, and explanatory factors discussed. Photos from SRMs and examples of data reviewed are available in S1 Fig. At each SRM, staff discussed performance against targets and made decisions regarding resource allocation, site-level target revisions, course correction, and technical strategies required to achieve project targets. Feedback was provided to sites through field visits, email, WhatsApp groups, and phone calls. Field visits were informed by data and were often to sites that were not meeting targets. During each visit, project staff helped frontline workers to review their performance against targets, identify root causes for underperformance, adopt and scale up best practices. SRM meetings continued from April 1 onwards, and have continued to date.

### Statistical analysis

We analyzed routinely collected program data from before and after the implementation of the improvement strategies and SRM meetings. The data are of program participants 16 years and older who sought HTS during the six months before the intervention (October 1, 2017–March 31, 2018) and 12 months after the intervention (April 1, 2018–March 31, 2019). Although some components of the intervention were rolled out over a few months, to simplify the analysis we treated April 1 as the beginning of all intervention components. To receive HTS, an individual must have self-identified as either an FSW, MSM or TG. Since the ODP specifically supports key populations, individuals who do not identify as such (for instance, clients of sex workers) are referred elsewhere; therefore, they are not included in this analysis.

An M&E officer at the ODP central office generated a dataset with the HTS data between the specified dates. HTS data were de-identified by removing participant names and unique IDs. The M&E officer then transferred the de-identified datafiles to the HQ-based analysis team for final cleaning and analyses.

To describe the overall sample characteristics, we calculated n’s, positivity rates, and case finding rates stratified by intervention period and KP type, age group, testing modality, and district. We compared the overall positivity rates for the 6 pre-intervention months to those of the 12 post-intervention months, explored the trends in the monthly positivity rates and case finding, and used a time series regression analysis to study the impact of the intervention. More specifically, we fitted a segmented linear regression model with a Newey-West variance estimator to account for the serial correlation between months [20]. We also fit this time series model by population type and testing modality. We excluded transgender population and facility testing modality from our models by population type and modality due to low sample sizes in some months. In fitting these models, we generally used a 1-month lag for the serial correlation structure; the serial correlation tests didn’t allow us to reject the null hypothesis the serial correlation in the time series died out after 1 month. We used Stata version 15 for all data management and analysis, and Linden’s user-contributed commands for conducting the interrupted time series analysis [21–23].

### Ethical review

Approval to analyse these data was obtained from FHI 360 Office of International Research Ethics (OIRE) and the ERES Converge IRB in Lusaka, Zambia. The study received a waiver of informed consent as the research involved no more than minimal risk to participants and could not be practicably carried out if informed consent was not waived. Approval to publish the manuscript was provided by the National Health Research Authority in Zambia (Ref No: NHRA00001/16/04/2021).

## Results

Between October 1, 2017 and March 31, 2019, 30,911 KP individuals (75.2% FSWs, 22.4% MSM, and 2.4% TG) 16 years and older received HTS—9,408 in the six months pre-intervention and 21,503 during the 12 months intervention period (Table 3). The mean age was 27 years. Table 3 shows the number of tests and HIV positivity rates for different KP groups, age categories, testing modalities, and by district before and after the intervention. Average monthly number of HIV tests increased 14% after the intervention, from an average of 1,568 individuals tested per month during pre-intervention to an average of 1,792 individuals tested per month during post-intervention. While it had the lowest positivity rate, the Community/Outreach testing modality reached the majority of patients (92.3% pre-intervention and 83.4% post-intervention) and found the majority of positive cases (86.7% pre-intervention, and 78.4% post-intervention).

**Table 3:** Participant characteristics and positivity by intervention period.

Characteristics	Pre-intervention (6 months)			Post-intervention (12 months)			Total
	*n*	*positivity*	*Case finding*	*n*	*positivity*	*Case finding*	*n*
KP group							
FSW	7,533	10.7%	806	15,724	36.0%	5,661	23,257
MSM	1,656	5.6%	93	5,266	22.5%	1,185	6,922
TG	219	5.0%	11	513	25.5%	131	732
Age group							
<18	771	2.2%	17	224	8.0%	18	995
18–24	4,021	5.6%	225	6028	18.2%	1,097	10049
24–29	2,352	11.2%	263	6166	29.9%	1,844	8518
29–34	1,274	15.9%	203	4381	38.7%	1,695	5655
34–39	586	19.5%	114	2614	48.5%	1,268	3200
39 or older	404	21.8%	88	2090	50.0%	1,045	2494
Testing modality							
Community/Outreach	8,684	8.9%	773	17,923	30.5%	5,467	26,607
Facility	724	19.3%	140	985	31.2%	307	1,709
Index/PNS	0	-		771	70.0%	540	771
SNS	0	-		1,824	35.9%	655	1,824
District							
Chililabombwe	1,224	7.4%	91	2,132	33.7%	718	3,356
Chirundu	890	4.6%	41	1,543	25.3%	390	2,434
Kabwe	963	5.8%	56	2,213	28.5%	631	3,177
Kapiri Mposhi	2,238	4.4%	98	2,051	27.3%	560	4,463
Kitwe	1,044	6.0%	63	3,833	25.6%	981	4,879
Livingstone	1,105	10.7%	118	2,557	39.9%	1,020	3,664
Lusaka	833	25.3%	211	4,457	35.2%	1,569	5,293
Solwezi	1,111	20.3%	226	2,717	40.3%	1,095	3,830
Total	9,408	9.5%	894	21,503	32.4%	6,967	30,911

**Fig 1:**
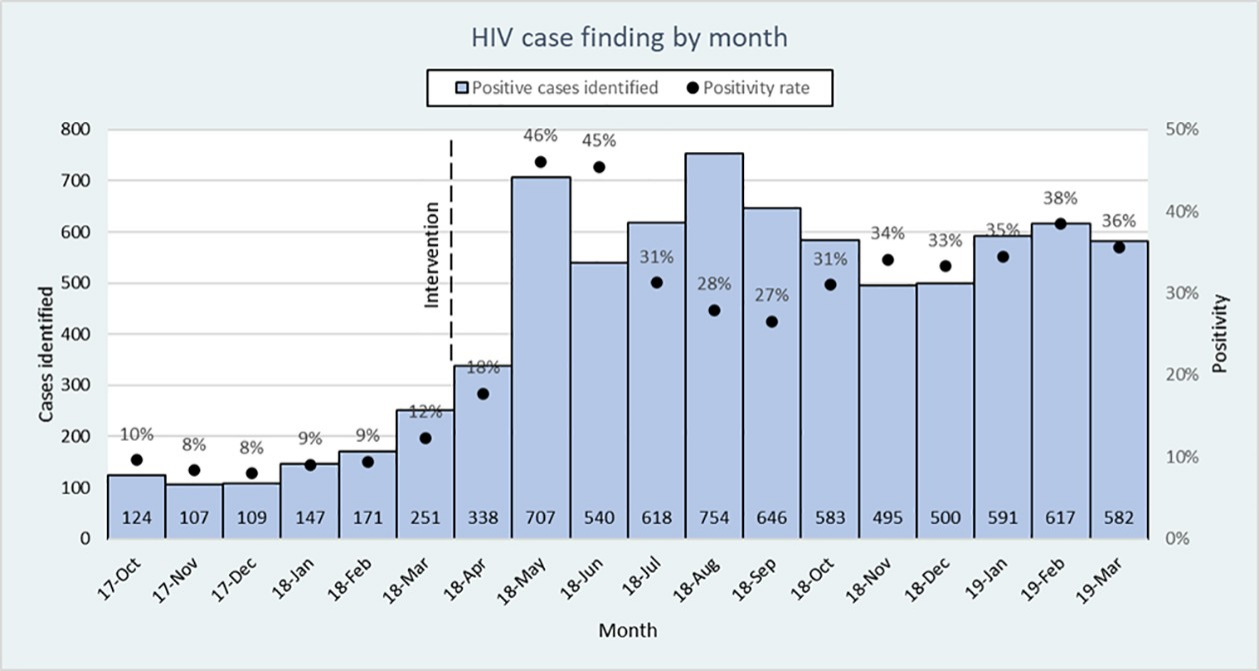
HIV case finding by month.

The positivity rate in the six pre-intervention months was 9.7% and increased to 32.4% during the 12 post-intervention months. Fig 1 shows the absolute number of cases identified per month. The monthly average number of positive cases identified went from 149 pre-intervention to 581 post-intervention, a 290% increase. Increases were also seen across all KP groups: from 10.7% to 36% among FSWs; 5.6% to 22.5% among MSM; and 5.0% to 25.5% among. Likewise, positivity rate increases were observed in all testing modalities and in each region, even in Lusaka where the pre-intervention positivity was already high at 25.3%.

**Fig 2:**
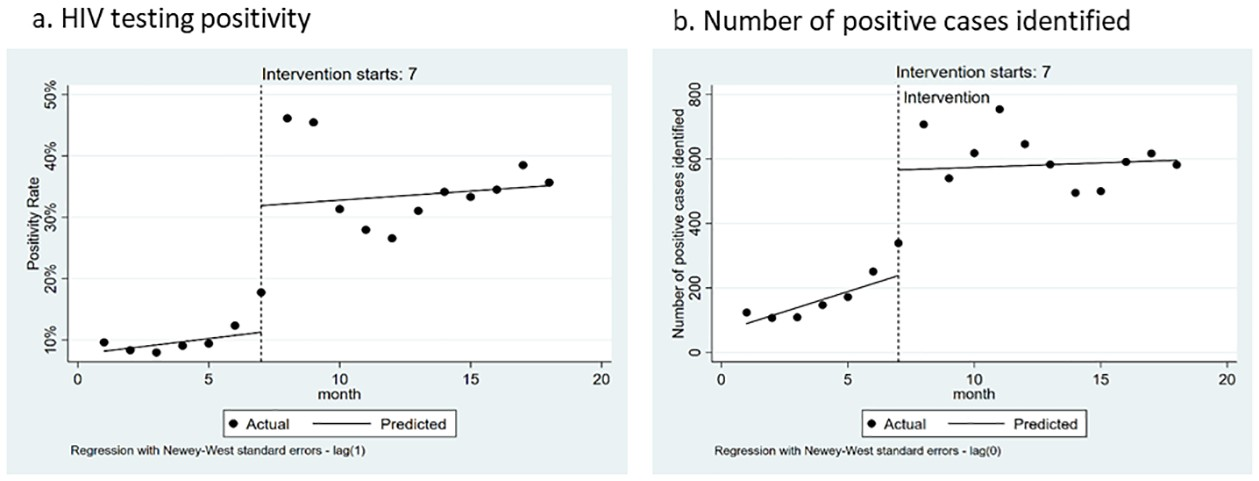
Percent of HIV tests that were positive and number of positive cases, by intervention period.

Based on the interrupted time series analysis (Table 4, Fig 2), the post-intervention monthly positivity increased by 20.6% (95% CI: 5.1%-36.1%, p = 0.01) and the monthly number of cases identified increased by 327 (95% CI:118.6–536.0, p = 0.005). Pre-intervention, case finding was increasing by 24.8 cases per month (95% CI: 6.6, 43.0; p = 0.01). Further, the slope of the post-intervention trend line did not differ statistically from the slope of the pre-intervention trend line.

**Table 4:** Results of interrupted time series analysis on positivity and case finding (n = 30,911).

	Coef.	Newey-West Std. Err.	p-value	95% CI
**Positivity rate**				
Pre-intervention trend	0.005	0.003	0.129	(-0.002, 0.013)
Effect of intervention	0.206	0.072	0.012	(0.052, 0.360)
Change in trend during intervention	-0.002	0.008	0.773	(-0.019, 0.014)
**Case finding**				
Pre-intervention trend	24.8	8.484	0.011	(6.6, 43.0)
Effect of intervention	327.3	97.307	0.005	(118.6, 536.0)
Change in trend during intervention	-22.0	14.336	0.147	(-52.8, 8.7)

**Fig 3:**
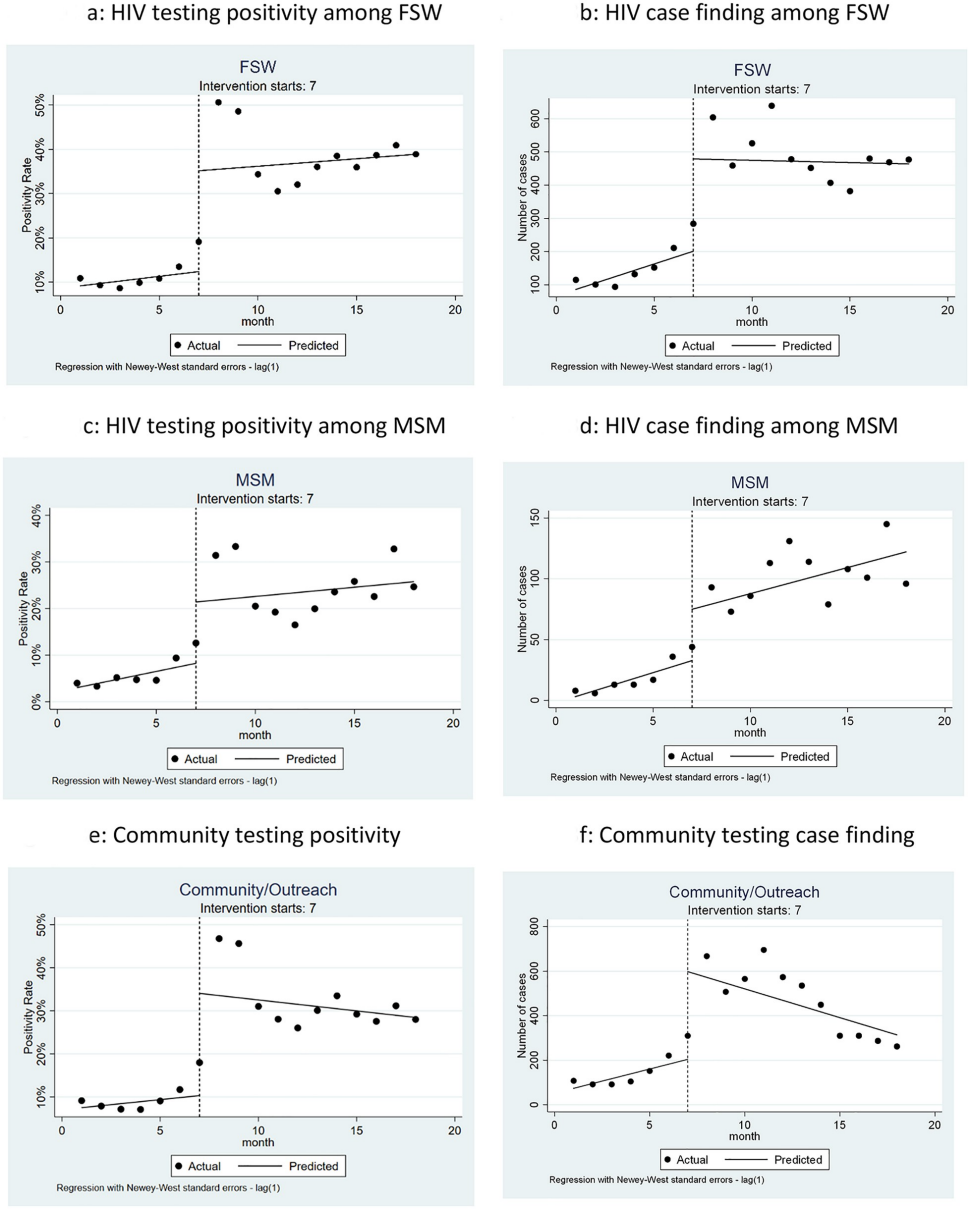
Percent of HIV tests that were positive and number of positive cases by population and testing modality.

We also analyzed changes in positivity and case finding by population and modality (Fig 3). Statistical results of these models are included in S1 Table. The immediate intervention effect shows statistically significant increases in positivity and case finding among both MSM and FSW, as well as among clients reached via community outreach. Positivity and case finding for both populations stay higher than pre-intervention rates throughout the intervention period. While improvements in community outreach positivity and case finding are maintained post-intervention, the trend in case finding post-intervention is negative. This can mostly be attributable to a large initial intervention effect that recedes over time.

## Discussion

To reach the 95-95-95 goals, programs must implement evidence-based interventions with fidelity to identify populations at risk, provide HTS, and link HIV-positive individuals to ART for sustained viral suppression. Results from this analysis of program data before and after implementation of managerial and technical interventions show that implementation of evidence-based interventions—implemented under an adaptive management approach focused on data use for decision-making—can lead to program improvement.

While resource constraints have led to an increased focus on improving testing efficiency, some stakeholders have cautioned that focusing on positivity rate as a primary criterion to measure success of HTS could lead to a decrease in the total number of positives identified [24]. In this analysis we have shown that programs can improve the positivity rate and more efficiently increase the total number of positive individuals identified simultaneously.

Other studies have shown that while SNS and index/PNS can lead to high positivity rates compared to other modalities, the absolute number of HIV-positive individuals identified is often low [6, 25, 26]. This is confirmed through this analysis, with only 17% of new positives being identified by SNS and index testing/PNS. This is ultimately due to the volume of clients reached through these approaches—only 12.1% of clients tested were reached through the new high yield modalities. Thus, a combination of testing approaches is critical, balancing the HIV positivity rate on one hand and reach/case finding on the other. In this program, community/outreach and facility testing, though achieving lower positivity rates compared to index/PNS and SNS, resulted in more HIV-positive KP individuals being identified. While the introduction of high yield testing modalities can explain some of the improvement in case finding, community/outreach and facility testing case finding rates also increased. This suggests that while the introduction of SNS and index/PNS were important components of success, other interventions focused on improving community outreach such as hotspot mapping and moonlighting also contributed to the impact of the intervention.

In ODP, we achieved higher positivity than other studies in Zambia and sub-Saharan Africa. A recent study of targeted testing strategies among the general population in Zambia showed positivity of index testing at 44.7% and other community testing (including mobile and testing at standalone VCT centres) at 23.2% [27]. A similar study in Zimbabwe found that index testing (32.6%) had much higher positivity than facility-based testing (4.1%) [28]. While positivity in our program was higher than these studies, some of the difference is likely attributed to our focus on KPs. In fact, a recent systematic review of testing positivity found that while there were only four studies specifically targeting KPs, those studies recorded higher positivity (24–55%), when compared to community-based general population (6–11%) or facility VCT (18–20%) testing strategies [29]. The success of KP projects in identifying new positive cases, and lack of published literature, suggests scale up of KP-focused strategies is urgently needed [30].

Involvement of target populations in planning and implementation is key in reaching unreached members of targeted populations [31–33]. The stigmatization and criminalization of many KPs make it increasingly important to garner acceptance among these populations. While KP partners were part of the project from the beginning, ODP directly involved KPs in the co-design of the managerial and technical interventions. Involving KPs helped the project identify gaps in the current approaches and decide to implement differentiated models of testing. Involvement of KPs in mobilizing peers for HIV services (EPOA), has been shown to be effective in identify unreached networks with HIV services [19, 34, 35]. This project successfully employed the approach to effectively reach KPs and achieved high positivity.

The intervention focused heavily on increased frequency of reporting and use of data to inform decisions, including at daily SRMs, an important aspect of TQLA. The use of data to inform program decisions is widely accepted, yet in many HIV programs more emphasis is placed on data collection than data use [36–38]. Recently, data use and increased monitoring has been promoted, with PEPFAR even recommending that many testing and treatment indicators be monitored on a weekly basis rather than the required reporting frequency of quarterly or semi-annually [11, 39]. While it is not possible to determine the role of intervention components singly, this analysis showed that implementing a combination of interventions, including daily reporting and continuous review of data and performance trends, allowed timely course correction and resulted in improvement over time.

This analysis, and the TQLA approach, have important limitations. Given the lack of an experimental design, observed changes in positivity rate cannot be attributed to TQLA, or specific management approaches or technical activities. It is not possible to determine whether the newly implemented technical approaches would have been successful without the new management approaches and daily data review, or vice versa. Further, the intervention components were introduced in a phased manner and often in combination making attribution of changes over time hard to determine. TQLA approaches such as convening meetings, investing in collection for daily data review, and daily targeted TA can be resource intensive. Cost data are not available to show if this approach or individual components are cost effective. Lastly, lack of accurate size estimation or prevalence data on different KPs makes it impossible to compare the case identification rate and positivity to the target populations as a whole.

Nonetheless, this analysis has many strengths. We used validated, individual level HIV testing data that allowed us to look at changes by covariates such as population type and site. Further, we analyzed data from six months before the intervention and 12 months after, allowing us not only to present a before and after scenario, but also to examine changes monthly over time. Often an intervention can provide an immediate improvement in the targeted outcome, yet over time that outcome comes back to pre-intervention levels. By including 12 post-intervention months, we were able to see that even after the initial improvement recedes, positivity rates settled into a more stable pattern, that is, even 12 months after the intervention, still significantly higher than positivity in all pre-intervention months.

## Conclusion

Implementing a targeted managerial and technical intervention in combination resulted in substantially greater HIV-testing positivity and case identification over the 12 months. These findings have important implications for HIV testing programs, especially those that target key populations, who often face access challenges because of criminalization and stigma. The ODP was able to reach large numbers of previously unreached KP individuals and identify those who were HIV positive, demonstrating that differentiated, data-driven, community-focused approaches can help close the 95-95-95 gaps.

## Supporting information

S1 Fig (PNG)

S1 Table (PDF)
