# Supplementary figures and images for: Improved HIV case finding among key populations after differentiated data driven community testing approaches in Zambia
Source: PLoS One. 2021 Dec 2;16(12):e0258573. doi: 10.1371/journal.pone.0258573 (PMC8638925; doi:10.1371/journal.pone.0258573)

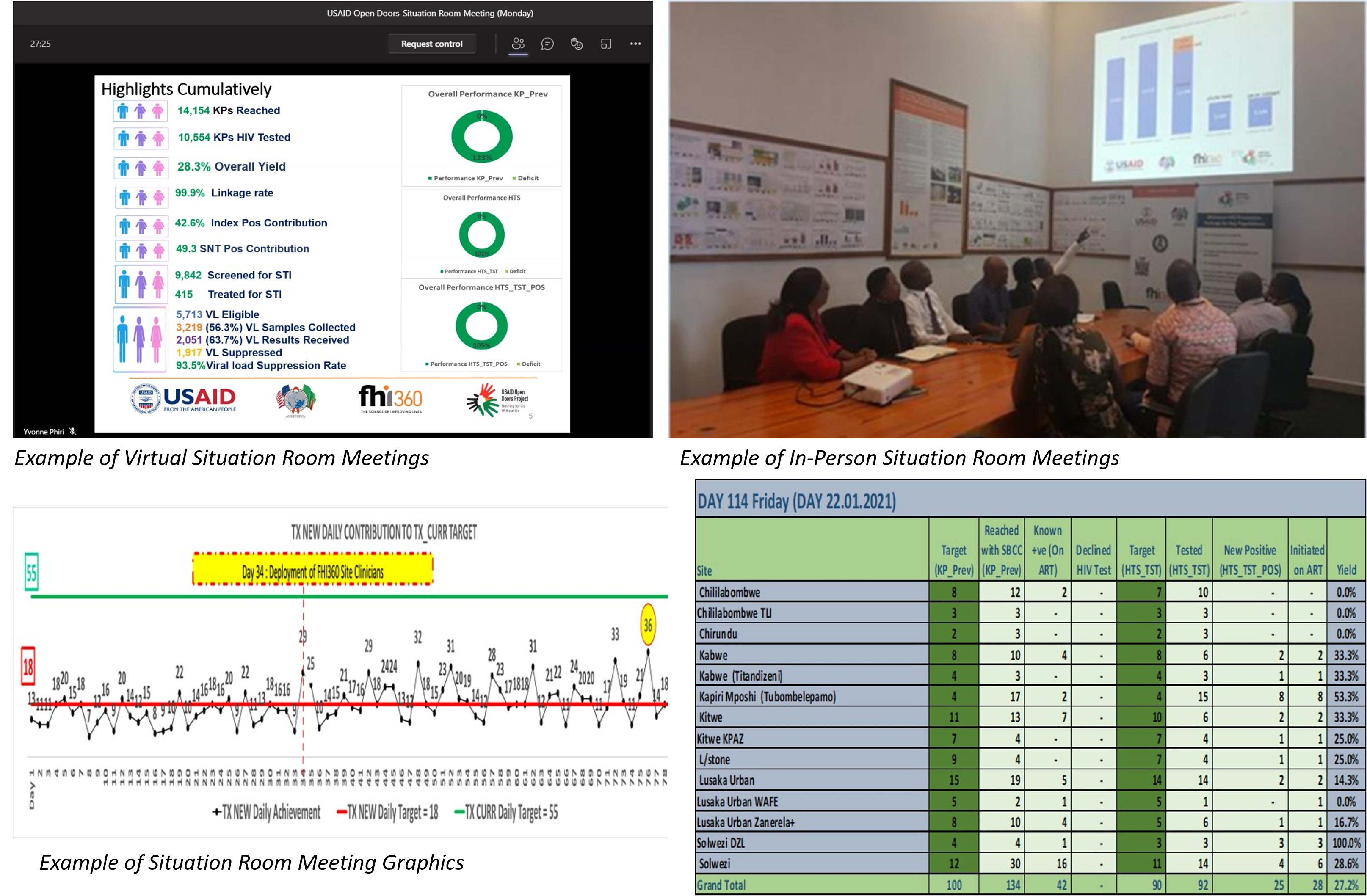

Supplement: S1 Fig — (PNG) [file pone.0258573.s001.png]
