# Supplementary material for: Improved HIV case finding among key populations after differentiated data driven community testing approaches in Zambia
Source: PLoS One. 2021 Dec 2;16(12):e0258573. doi: 10.1371/journal.pone.0258573 (PMC8638925; doi:10.1371/journal.pone.0258573)
Supplement: S1 Table — (PDF) [file pone.0258573.s002.pdf]

S2a: Results of Interrupted Time Series Analysis on Positivity and Case finding among FSW

**Positivity**	Coef.	Newey-West Std. Err.	p-value	95% CI	
Pre-intervention trend	0.005368	0.003651	0.164	-0.00246	0.013198
Effect of intervention	0.227772	0.076117	0.01	0.064518	0.391026
Change in trend during intervention	-0.00198	0.008208	0.813	-0.01958	0.015624
**Cases**					
Pre-intervention trend	19.17143	6.288868	0.009	5.683149	32.65971
Effect of intervention	277.5923	78.08992	0.003	110.1061	445.0785
Change in trend during intervention	-20.5246	7.955951	0.022	-37.5884	-3.46076

S2b: Results of Interrupted Time Series Analysis on Positivity and Case finding among MSM

**Positivity**	Coef.	Newey-West Std. Err.	p-value	P>|t|	95% CI	
Pre-intervention trend	0.00869	0.00324	2.68	0.018	0.001744	0.01564
Effect of intervention	0.13161	0.05628	2.34	0.035	0.010912	0.252314
Change in trend during intervention	- 0.00474	0.00596	-0.8	0.44	-0.01751	0.008036
**Cases**						
Pre-intervention trend	4.9428	1.38968	3.56	0.003	1.962283	7.923431
Effect of intervention	42.1872	15.3520	2.75	0.016	9.260386	75.11397
Change in trend during intervention	- 0.65265	1.62004	-0.4	0.693	-4.12721	2.821915

S2c: Results of Interrupted Time Series Analysis on Positivity and Case finding among cases identified through Community/Outreach testing

**Positivity**	Coef.	Newey-West Std. Err.	p-value	P>|t|	95% CI	
Pre-intervention trend	0.004672	0.003946	1.18	0.256	-0.00379	0.013135
Effect of intervention	0.237462	0.074512	3.19	0.007	0.077651	0.397274
Change in trend during intervention	-0.00976	0.007847	-1.24	0.234	-0.02659	0.007069
**Cases**						
Pre-intervention trend	21.65714	8.041328	2.69	0.017	4.410209	38.90408
Effect of intervention	393.5846	114.8802	3.43	0.004	147.1911	639.9782
Change in trend during intervention	-47.4544	11.91529	-3.98	0.001	-73.0101	-21.8986
